# β,β-Dimethylacrylshikonin Induces Mitochondria Dependent Apoptosis through ERK Pathway in Human Gastric Cancer SGC-7901 Cells

**DOI:** 10.1371/journal.pone.0041773

**Published:** 2012-07-27

**Authors:** Xiu-Jin Shen, Hai-Bing Wang, Xiao-Qiong Ma, Jiang-Hua Chen

**Affiliations:** 1 Kidney Disease Center, The First Affiliated Hospital, College of Medicine, Zhejiang University, Hangzhou, China; 2 National Clinical Research Base of Traditional Chinese Medicine, Zhejiang Hospital of Traditional Chinese Medicine, Zhejiang Chinese Medical University, Hangzhou, China; Duke University Medical Center, United States of America

## Abstract

β,β-Dimethylacrylshikonin, one of the active components in the root extracts of Lithospermum erythrorhizon, posses antitumor activity. In this study, we discussed the molecular mechanisms of β,β-dimethylacrylshikonin in the apoptosis of SGC-7901 cells. β,β-Dimethylacrylshikonin reduced the cell viability of SGC-7901 cells in a dose- and time-dependent manner and induced cell apoptosis. β,β-Dimethylacrylshikonin treatment in SGC-7901 cells down-regulated the expression of XIAP, cIAP-2, and Bcl-2 and up-regulated the expression of Bak and Bax and caused the loss of mitochondrial membrane potential and release of cytochrome c. Additionally, β,β-dimethylacrylshikonin treatment led to activation of caspases-9, 8 and 3, and cleavage of poly (ADP-ribose) polymerase (PARP), which was abolished by pretreatment with the pan-caspase inhibitor Z-VAD-FMK. β,β-Dimethylacrylshikonin induced phosphorylation of extracellular signal-regulated kinase (ERK) in SGC-7901 cells. U0126, a specific MEK inhibitor, blocked the ERK activation by β,β-dimethylacrylshikonin and abrogated β,β-dimethylacrylshikonin -induced apoptosis. Our results demonstrated that β,β-dimethylacrylshikonin inhibited growth of gastric cancer SGC-7901 cells by inducing ERK signaling pathway, and provided a clue for preclinical and clinical evaluation of β,β-dimethylacrylshikonin for gastric cancer therapy.

## Introduction

Gastric cancer is one of the aggressive malignant tumors, although with the development of radiotherapy, chemotherapy and biotherapy, the severe side effects are unavoidable [1], therefore, more effective antitumor drugs with fewer side effects for the treatment of gastric cancer are needed.

**Figure 1:**
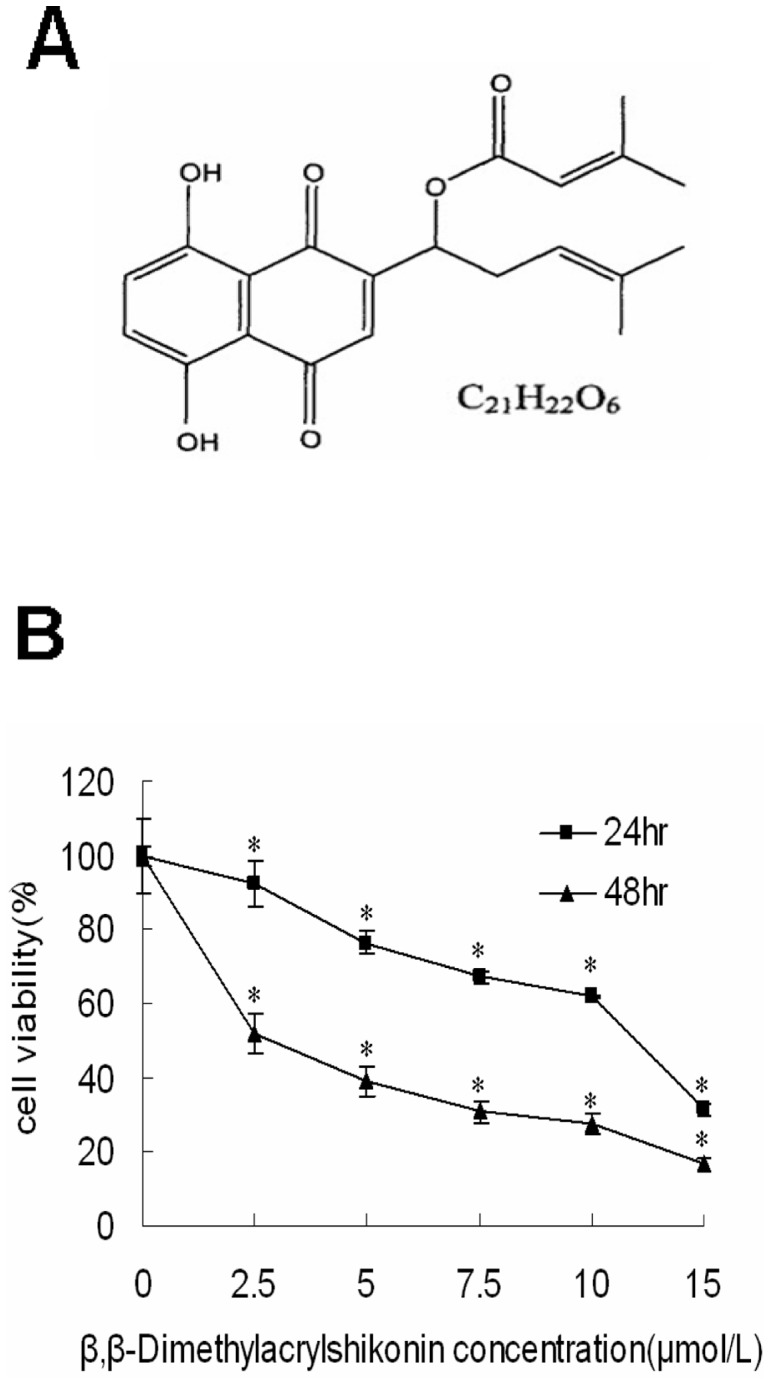
β,β-Dimethylacrylshikonin inhibits SGC-7901 cells viability. (A) The chemical structure of β,β-dimethylacrylshikonin (MW. 370.4). (B) Effects of β,β-dimethylacrylshikonin on cell viability inhibition of SGC-7901 cells. Cells were treated with different concentrations of β,β-dimethylacrylshikonin for 24 h and 48 h. The related cell viability was determined by MTT assay. The viability of the control group (0.1% DMSO) was set to 100%. Data represent the mean±SD obtained from three independent experiments. * indicated p<0.05 compared to control group respectively.

Lithospermum erythrorhizon is an important Chinese herb. It has some active components: Deoxyshikonin, Isobutyrylshikonin, Acetylshikonin, Isovalerylshikonin, β,β-Dimethylaerylshikonin(Fig. 1A) and shikonin [2]. In traditional Chinese medicine, it exhibits multiple biological functions including anti-microbial, anti-inflammatory, anti-tumor, immune regulation and anti-HIV properties [3]–[6]. The anti-tumor effect of shikonin and its derivatives were first proved by their activities against tumor growth in murine Sarcoma-180 [7]. Shikonin exhibits effect not merely by killing tumor cells directly, but also by inhibiting tumor angiogenesis [8]. Studies revealed that shikonin induced apoptosis in human malignant melanoma, bladder cancer, cervical cancer, lung cancer and liver cancer, and so on [9]–[13]. However, it has less side effects and more protective effects on human normal cells [14], [15]. Hsu PC et al showed that shikonin led to cell apoptosis through up-regulation of p27, p53, Bax and down-regulation of Bcl-2 and Bcl-xL in human colorectal carcinoma COLO 205 cells [16]. The tumor invasion inhibited by shikonin in some cancer cells may through the down-regulation of NF-κB-mediated MMP-9 expression [17]. Shikonin also induces apoptosis via ROS production in hepatocellular carcinoma [12]. Singh F et al also found that Shikonin decreased phosphorylated levels of EGFR, ERK and protein tyrosine kinases and increased intracellular levels of apoptosis-related proteins, which caused epidermoid carcinoma cells to undergo apoptosis [18].

Recent evidence showed that β,β-dimethylacrylshikonin had significant anti-tumor effect on hepatocellular carcinoma by activating caspase-3 [19]. However, such effect of β,β-dimethylacrylshikonin on human gastric cancer cells has not been reported and the molecular mechanisms are still not well understood. Thus, in the present study, We will discuss effect of β,β-dimethylacrylshikonin on human gastric cancer cell SGC-7901 and its related signaling to better understand the mechanism of β,β-dimethylacrylshikonin on gastric cancer.

## Materials and Methods

### Materials and Reagents

SGC-7901 cells were purchased from Chinese Academy of Sciences Cell Bank of Type Culture Collection (Shanghai, China). β,β-Dimethylacrylshikonin was purchased from Tokyo Chemical Industry (Tokyo, Japan). MTT and DAPI were purchased from Calbiochem (San Diego, CA, USA). U0126 was purchased from Cell Signaling (Boston, MA, USA). Pan-caspase inhibitor (Z-VAD-FMK) was purchased from Beyotime Institute of Biotechnology (Shanghai, China). Antibody for cytochrome c was purchased from Santa Cruz Biotechnology (Santa Cruz, CA, USA). Antibodies against ERK, phospho-ERK, Bcl-2, Bcl-xl, XIAP, cIAP-2, survivn, Bax, Bak, cleaved caspase-9, cleaved caspase-3, cleaved PARP and β-actin were purchased from Cell Signaling (Boston, MA, USA). FITC-Annexin V Apoptosis Detection Kit was purchased from Becton Dickinson (San Diego, CA, USA).

### Cell culture and cell proliferation assay

SGC-7901 cells were cultured in RPMI 1640 medium with 10% fetal bovine serum (Hyclone, UT), and maintained at 37°C in a humidified atmosphere of 5% CO_2_. Then cells were seeded in a 96-well dish to a final concentration of 5×10^3^ cells/well and incubated in RPMI 1640 medium containing 10% FCS for 24 h, after that, cells were treated with the indicated concentration of β,β-dimethylacrylshikonin for 24 h and 48 h. Medium was removed and fresh medium was added to each well along with 20 µl of MTT solution (5 mg/ml). After 4 h incubation, 150 µl of DMSO was added to each well. The plates were read at wavelength of 570 nm using Varioskan Flash Multimode Reader (Thermo Scientific, USA). Four reduplicate wells were used for each treatment, and experiments were repeated three times.

### Morphological changes

SGC-7901 cells were placed in the well of a six-well plate. After 24 h cell culture, they were treated with β,β-dimethylacrylshikonin for the indicated time periods. The cellular morphology was observed using an inverted microscopy (model IX70; Olympus, Tokyo, Japan).

### DAPI (4′, 6-diamidino-2-phenylindole) staining

SGC-7901 cells were placed in the well of a six-well plate. After 24 h cell culture, they were treated with β,β-dimethylacrylshikonin for the indicated time periods, then cells were fixed in cold acetone for 30 min, and incubated with DAPI (1 mg/ml) for 30 min. The apoptotic nuclei characterized as intensively stained were detected using fluorescent microscopy (model IX71; Olympus, Tokyo, Japan).

### Annexin V/PI assays for apoptosis

For Annexin V/PI assays, cells were stained with AnnexinV-FITC and PI, and evaluated for apoptosis by flow cytometry according to the mannufacturer's protocol (BD PharMingen, San Diego, CA, USA). Briefly, 1×10^5^ cells were washed twice with PBS, and stained with 5 µl of AnnexinV-FITC and 5 µl of PI in 500 µl binding buffer for 15 min at room temperature in the dark. The apoptotic cells were determined using BD FACS Diva software (BD Biosciences, Franklin Lakes, NJ). Annexin V staining serves as a measure of phosphatidylserine externalization and cells that are Annexin V^+^/PI^−^ represent early apoptotic cells.

### Measurement of mitochondrial membrane potential

Cells (5×10^5^ cells/ml) were treated with or without β,β-dimethylacrylshikonin (10 µmol/L) for 24 h and stained with JC-1(BD MitoScreen JC-1 Kit, Becton Dickinson, USA) for 15 min at 37°C. Mitochondrial membrane potential was detected by flow cytometry (FACSCanto II, Becton Dickinson, USA).

### Western Bloting analysis

Cells were treated with the indicated concentration of β,β-dimethylacrylshikonin for the indicated time in RPMI 1640 medium with 10% FCS. The cells were collected in ice-cold PBS, and the cell extracts were prepared in RIPA buffer with proteinase inhibitor cocktail from Calbiochem (San Diego, CA). The protein concentrations of the cell lysates were determined and boiled with gel-loading buffer for 10 min at 100°C. Samples containing 30 µg of total protein were electrophoresed on 10% SDS-polyacrylamide gels and transferred to PVDF membrane (Millipore, Temecula, CA). Following transfer, the membrane were blocked in TBST (TBS containing 0.1% Tween 20) containing 5% skimmed milk for 2 h, followed by incubation overnight at 4°C with appropriate primary antibodies. After washing three times in TBST, the membranes were incubated for 2 h at 37°C with 1∶5000 horseradish peroxidase-conjugated appropriate secondary antibodies. Finally, the membranes were visualized using the enhanced chemiluminescence detection system (Immun-Star WesternC Kit, Bio-Rad, USA).

### Immunofluorescence staining

Immunofluorescene staining was used to analyze the subcellular distribution of cytochrome c in SGC-7901 cells induced by β,β-dimethylacrylshikonin. Cells cultured on sterile glass coverlips were fixed in cold acetone for 10 min. After permeabilized with 0.3% Triton X-100 for 20 min at room temperature, cells were blocked in 3% bovine serum albumin for 30 min and incubated overnight at 4°C with anti-cytochrome c antibody(1∶30). After washing three times in PBS, SGC-7901 cells were labeled with Alexa Fluor 488-conjugated secondary antibody. DAPI was subsequently added for nuclear staining. Microscopic analysis was performed using a fluorescent microscopy (model IX71; Olympus, Tokyo, Japan).

### Statistical analysis

Data reported are the mean±standard deviation (SD) of three independent experiments. They were evaluated by one-way analysis of variance (ANOVA). Significant differences were established at p<0.05.

## Results

### β,β-Dimethylacrylshikonin inhibits viability of SGC-7901 cells

In order to determine the cytotoxic effect of β,β-dimethylacrylshikonin on gastric cancer cells. SGC-7901 cells were treated with 2.5, 5, 7.5, 10 µmol/L of β,β-dimethylacrylshikonin for 24 h and 48 h, the reduced viability of cells treated with β,β-dimethylacrylshikonin was 92.41±6.06%, 76.49±3.18%, 67.19±1.82%, and 61.76±0.18% respectively at 24 h compared with the vehicle-treated cells(Fig. 1B). For the 48 h treatment, cell viability was 52.03±9.45%, 38.99±1.43%, 30.70±1.58%, and 27.4±0.31% (Fig. 1B). The half maximal inhibitory concentration (IC_50_) of β,β-dimethylacrylshikonin for SGC-7901 cells was 11.04 µM and 2.89 µM at 24 h and 48 h respectively.

### β,β-Dimethylacrylshikonin induces SGC-7901 cells apoptosis

**Figure 2:**
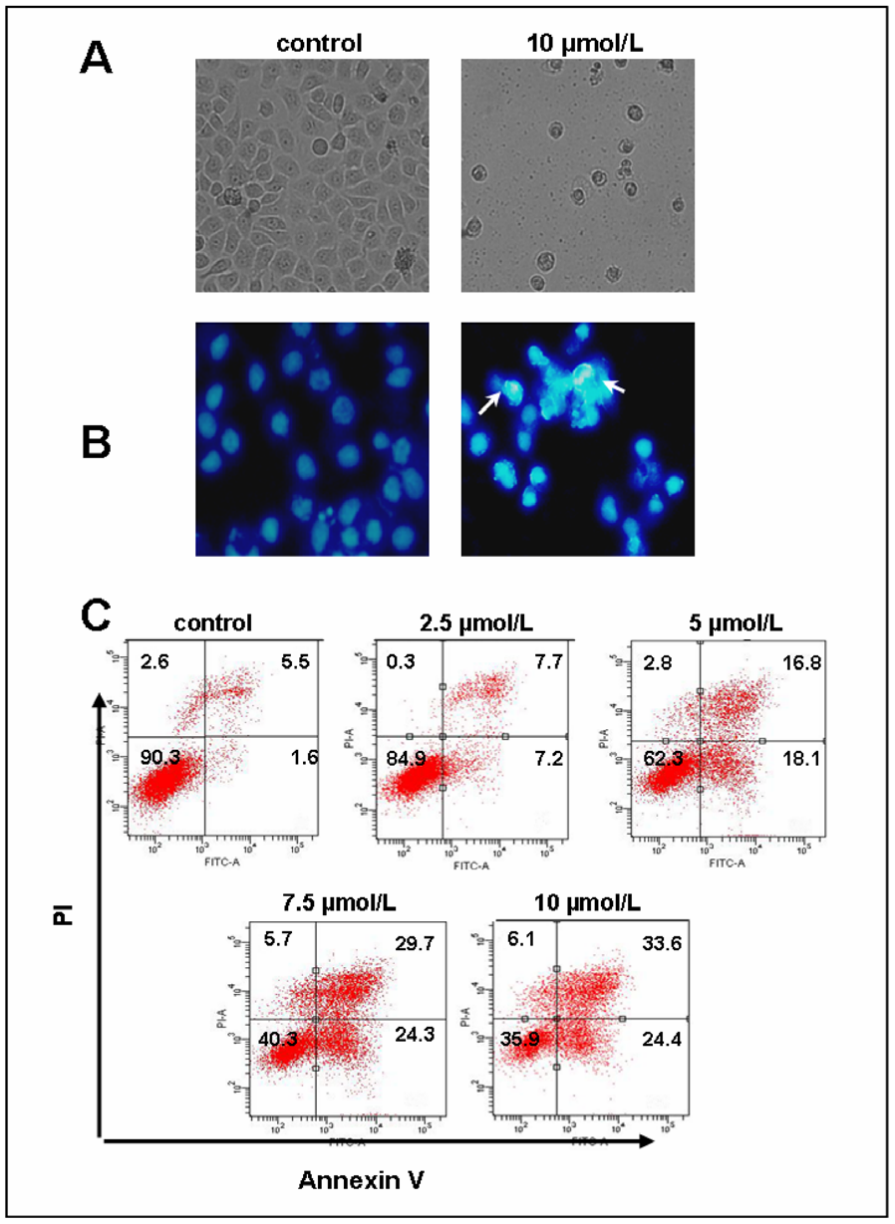
β,β-Dimethylacrylshikonin induced apoptosis in SGC-7901 cells. (A) Cell morphology was detected by using an invert microscope with 200×magnification. (B) DNA condensation (white arrow) was measured by DAPI stain and analyzed under a fluorescent microscope with 200×magnification. (C) The apoptotic status was evaluated by Annexin V-FITC binding assay. The lower right part (Annexin V-FITC^+^/PI^−^) was considered as early stage of apoptotic cells and top right part (Annexin V-FITC^+^/PI^+^) was considered as late stage of apoptotic cells. The lower left part (Annexin V-FITC^−^/PI^−^) was considered as viable cells and the upper left part (Annexin V-FITC^−^/PI^+^) was considered as necrotic cells.

SGC-7901 cells were first treated with β,β-dimethylacrylshikonin, results showed the cells underwent marked morphological changes upon treatment with 10 µmol/L β,β-dimethylacrylshikonin compared with the untreated control for 24 h. In the presence of β,β-dimethylacrylshikonin, SGC-7901 cell number reduced and the cells became round up and small in shape, and detaching from the plate compared with the negative control group (Fig. 2A). β,β-dimethylacrylshikonin also induced nuclear condensation with intensive DAPI staining compared to the nuclear of control SGC-7901 cells for 24 h (Fig. 2B).

To determine whether cytotoxity of β,β-dimethylacrylshikonin involved apoptosis, we evaluated the apoptotic effect of β,β-dimethylacrylshikonin in SGC-7901 cells by flow cytometry for Annexin V and propidium iodide(PI) staining. The percentage of early apoptotic cells (Annexin V^+^/PI^−^) was 1.6%, 7.2%, 18.1%, 24.3%, and 24.4% in response to the vehicle, 2.5, 5, 7.5, and 10 µmol/L β,β-dimethylacrylshikonin respectively for 24 h (Fig. 2C). Interestingly, The late apoptotic cells (Annexin V^+^/PI^+^) were significantly increased in SGC-7901 cells (29.7% with 7.5 µmol/L and 33.6% with 10 µmol/L β,β-dimethylacrylshikonin treatment) (Fig. 2C).

### β,β-dimethylacrylshikonin induces mitochondrial events associated with apoptosis in SGC-7901 cells

**Figure 3:**
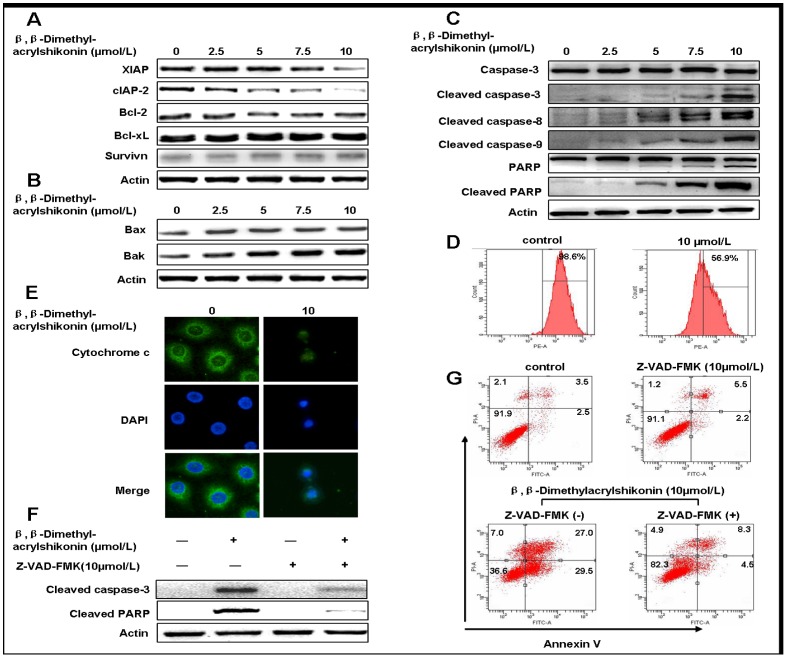
β,β-Dimethylacrylshikonin induced mitochondrial events associated with apoptosis in SGC-7901 cells. Western blotting analysis for anti-apoptotic proteins: Bcl-2, Bcl-xL, cIAP-2, XIAP and survivin(A), and pro-apoptotic proteins: Bax, Bak (B), and cleaved PARP, cleaved caspase-9, cleaved caspase-8, cleaved caspase-3(C) in whole cell extracts after the SGC-7901 cells were treated with β,β-dimethylacrylshikonin for 24 h. Protein levels of actin were also measured as controls. (D) Detection of mitochondrial membrane potential by flow cytometry. SGC-7901 cells were treated with or without β,β-dimethylacrylshikonin (10 µmol/L) for 24 h and were stained with JC-1 for 15 min at 37°C and subjected to flow cytometry. (E) SGC-7901 cells were fixed and labeled for cytochrome c (green) and DNA (blue). (F) SGC-7901 cells were treated with β,β-dimethylacrylshikonin in the presence or absence of Z-VAD-FMK (10 µmol/L) for 24 h. Protein extracts were prepared and subjected to Western blot assay using antibody against cleaved caspase-3, cleaved PARP. Protein levels of actin were also measured as controls. (G) SGC-7901 cells were treated with β,β-dimethylacrylshikonin in the presence or absence of Z-VAD-FMK (10 µmol/L) for 24 h. The apoptotic status was determined by Annexin V-FITC binding assay. The lower right part (Annexin V-FITC^+^/PI^−^) was considered as early stage of apoptotic cells and top right part (Annexin V-FITC^+^/PI^+^) was considered as late stage of apoptotic cells. The lower left part (Annexin V-FITC^−^/PI^−^) was considered as viable cells and the upper left part (Annexin V-FITC^−^/PI^+^) was considered as necrotic cells.

Bcl-2 family proteins include pro-apoptotic proteins (Bax, Bak and Bid) and anti-apoptotic proteins (Bcl-2, Bcl-xL, cIAP-2, XIAP and survivin). They can activate or inhibit the release of downstream factors which lead to the activation of caspase-3 and PARP in the execution of apoptosis [20]. In order to detect the apoptosis-related proteins, pro-apoptotic proteins (Bax, Bak) and anti-apoptotic proteins (XIAP, cIAP-2, Bcl-2, Bcl-xL, survivin) were detected by Western blotting after SGC-7901 cells were treated with different concentration of β,β-dimethylacrylshikonin (0, 2.5, 5, 7.5, and 10 µmol/L) for 24 h. The results indicated that β,β-dimethylacrylshikonin decrease the expression of XIAP, cIAP-2, Bcl-2 (Fig. 3A). However, the down-regulation of XIAP and Bcl-2 were less pronounced while the down-regulation of cIAP-2 was quite dramatic and dose-dependent. There was no significant change of Bcl-xL and survivn in SGC-7901 cells. Whether β,β-dimethylacrylshikonin can modulate the expression of pro-apoptotic proteins (Bax, Bak) was also examined. We found that β,β-dimethylacrylshikonin increased the expression of Bak in a dose-dependent manner but had little effect on Bax (Fig. 3B).

Caspase family proteins are critical enzymes to execute apoptosis. Among caspase family members, caspase-3 is a key executioner for apoptosis in mammalian cells [21]. It can be activated through death receptor-mediated extrinsic caspase-8 pathway and the mitochondria dependent-caspase-9 intrinsic pathway [22], [23]. In order to further understand the molecular mechanism of the apoptosis, SGC-7901 cells were treated with β,β-dimethylacrylshikonin and detected by Western blotting analysis for the expression change of caspase-3, caspase-8 and caspase-9, results showed a dose-dependent elevation of cleaved caspase-3, cleaved caspase-8 and cleaved caspase-9 in β,β-dimethylacrylshikonin treated cells. PARP cleavage, another well-known characteristic of apoptosis, was also found in β,β-dimethylacrylshikonin treated cells(Fig. 3C). Mitochondrial dysfunction induced apoptosis which is often the consequence of a decrease of mitochondrial membrane potential. It has been shown to be central to the apoptotic pathway. The SGC-7901 cells were treated with or without β,β-dimethylacrylshikonin (10 µmol/L) for 24 h, results showed mitochondria membrane potential was decreased from 98.6% to 56.9% after β,β-dimethylacrylshikonin was treated to the SGC-7901 cells (Fig. 3D). We next examined the distribution and subcellular localization of cytochrome c to find whether the mitochondria pathway is involved in β,β-dimethylacrylshikonin induced apoptosis. After SGC-7901 cells were treated with β,β-dimethylacrylshikonin (10 µmol/L) for 24 h, the distribution of cytochrome c was visualized with a confocal laser microscope, the β,β-dimethylacrylshikonin treated cells showed blurred morphology (Fig. 3E) in contrast to the obviously clear appearance in the control cells, indicating the translocation of cytochrome c from the mitochondria into the cytoplasm in β,β-dimethylacrylshikonin treated cells.

To further determine the role of caspase activation in β,β-dimethylacrylshikonin- induced apoptosis, we treated SGC-7901 cells with pan-caspase inhibitor Z-VAD-FMK(10 µmol/L) before β,β-dimethylacrylshikonin treatment. The pan-caspase inhibitor Z-VAD-FMK pretreatment decreased the expression of cleaved caspase-3, cleaved PARP and reduced β,β-dimethylacrylshikonin-induced apoptosis (Fig. 3F & G). These data showed that mitochondrial-mediated caspase cascade pathway plays a very important role in β,β-dimethylacrylshikonin-induced apoptosis.

### β,β-Dimethylacrylshikonin induces sustained ERK activation in SGC-7901 cells

**Figure 4:**
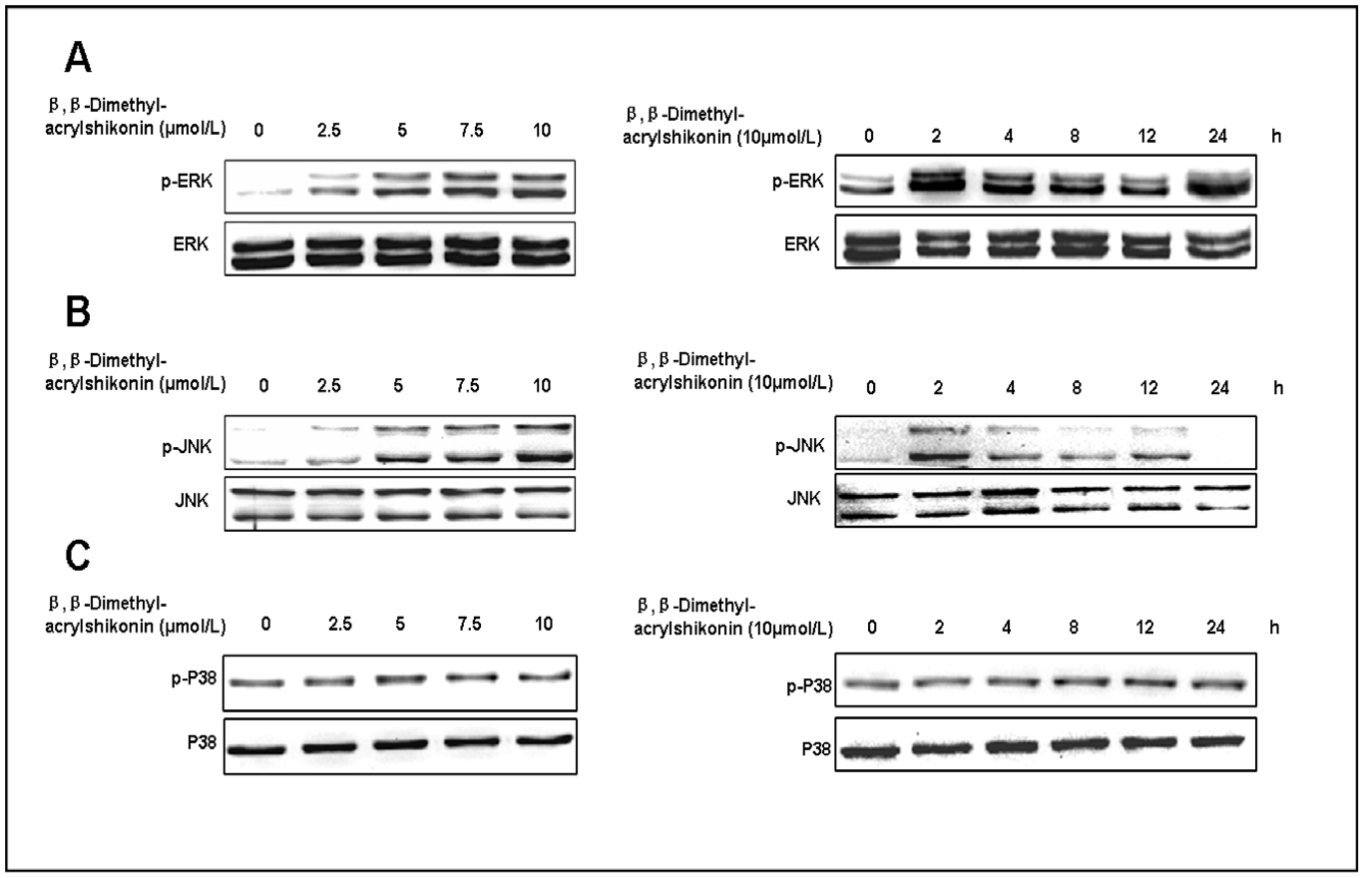
Effects of β,β-Dimethylacrylshikonin on MAPK pathways. SGC-7901 cells were treated with the indicated β,β-dimethylacrylshikonin concentration or the indicated interval, total cellular extracts were prepared and subjected to Western blot assay to measure levels of phosphorylated forms of ERK(A), JNK(B) and p38(C). Membranes were reprobed with antibodies against total ERK, JNK and p38 for normalization.

It was shown that MAPK signaling involved in several events of cellular stresses and stimuli induced cell apoptosis [24], [25]. We therefore examined the activation of ERK, JNK and p38 after β,β-dimethylacrylshikonin treatment. As shown in Fig. 4A & B, the phosphorylation levels of ERK and JNK were apparently increased in response to the β,β-dimethylacrylshikonin treatment for 2 h, and the phosphorylation levels exhibited a dose-dependent manner. Besides, the phosphorylation levels of ERK last twenty-four hours. However, no significant changes of phosphorylation levels of p38 were observed after the β,β-dimethylacrylshikonin treatment (Fig. 4C). These results suggested that sustained activation of the ERK is involved in β,β-dimethylacrylshikonin-induced apoptosis in SGC-7901 cells.

### The ERK signaling pathway is involved in β,β-dimethylacrylshikonin induced apoptosis in SGC-7901 cells

**Figure 5:**
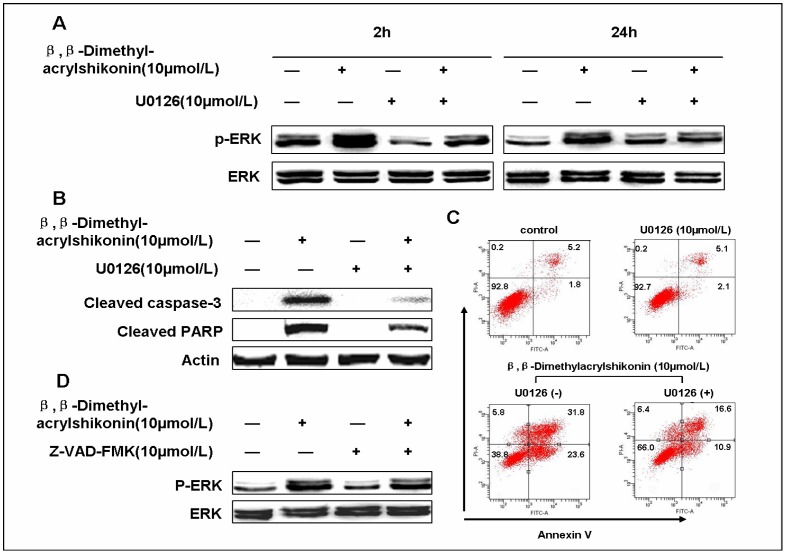
β,β-Dimethylacrylshikonin induced SGC-7901 cells apoptosis was mediated through ERK activation. (A) SGC-7901 cells were pretreated or not pretreated with U0126 (10 µmol/L) then added with DMSO (vehicle) or β,β-dimethylacrylshikonin (10 µmol/L) for 2 h, 24 h respectively. Protein extracts were prepared and subjected to Western blot assay to measure levels of phosphorylated ERK. Protein levels of total ERK were also measure as controls. (B) SGC-7901 cells were pretreated or not pretreated with U0126 (10 µmol/L) then added with DMSO (vehicle) or β,β-dimethylacrylshikonin (10 µmol/L) for 24 h. Protein extracts were prepared and subjected to Western blot assay using antibody against cleaved caspase-3, cleaved PARP. Protein levels of actin were also measured as controls. (C) SGC-7901 cells were treated with β,β-dimethylacrylshikonin in the presence or absence of U0126 (10 µmol/L) for 24 h. The apoptotic status was determined by Annexin V-FITC binding assay. The lower right part (Annexin V-FITC^+^/PI^−^) was considered as early stage of apoptotic cells and top right part (Annexin V-FITC^+^/PI^+^) was considered as late stage of apoptotic cells. The lower left part (Annexin V-FITC^−^/PI^−^) was considered as viable cells and the upper left part (Annexin V-FITC^−^/PI^+^) was considered as necrotic cells. (D) SGC-7901 cells were pretreated with or without Z-VAD-FMK (10 µmol/L) were further incubated with β,β-dimethylacrylshikonin (10 µmol/L) for 24 h. Protein extracts were prepared and subjected to Western blot assay to measure levels of phosphorylated ERK. Protein levels of ERK were also measured as controls.

We then examined whether the β,β-dimethylacrylshikonin-induced sustained activation of the ERK signaling pathway plays a role in apoptosis. As shown in Fig. 5A & B, Western blot assay revealed that U0126 (a specific MEK inhibitor) inhibited sustained ERK activation and cleavage of caspase-3, PARP in β,β-dimethylacrylshikonin treatment while the pan-caspase inhibitor Z-VAD-FMK had no effect on β,β-dimethylacrylshikonin-induced activation of ERK (Fig. 5D). In addition, U0126 reduced β,β-dimethylacrylshikonin-induced apoptosis in SGC-7901 cells (Fig. 5C). These results showed that β,β-dimethylacrylshikonin-induced apoptosis in SGC-7901 cells are mediated by the sustained activation of the ERK signaling pathway.

## Discussion

Shikonin derivatives are the active components isolated from the Chinese herbal Lithospermum erythrorhizon [5]. These compounds are promising drug candidates as they have been reported to have multiple anticancer effects in vivo and in vitro [5], [26]. Among the components of Lithospermum erythrorhizon, β,β-Dimethylaerylshikonin has important anti-tumor effects compared to other active ingredients [27]. Regarding to anti-cancer activity, shikonin-rendered cell apoptosis through activation of caspase-dependent pathway was found in many malignant tumors. In this study, we investigated the effect of β,β-dimethylacrylshikonin on human gastric cancer SGC-7901 cells. Our results indicated that treatment of SGC-7901 cells with β,β-dimethylacrylshikonin showed a significant cytotoxic effect against SGC-7901 cells in a dose and time dependent manner, and β,β-dimethylacrylshikonin induced apoptosis in SGC-7901 cells was evidenced by an increased early apoptotic cells (Annexin V^+^/PI^−^) and late apoptotic cells (Annexin V^+^/PI^+^).

The ratio of anti- and pro-apoptotic protein expression, such as Bcl-2/Bax, is crucial for the induction of apoptosis, and it decides the susceptibility of cells to undergo apoptosis [28]. Mitochondria play an important role in the signal transduction of apoptosis [29]. The translocalization of apoptotic proteins from the cytosol to the mitochondria leads to the release of cytochrome c and second mitochondria-derived activator of caspases by a decrease in mitochondrial membrane potential [30], [31]. Shikonin has been reported to induce apoptosis via mitochondria pathway in HepG2 cells [32]. In the present study, we showed that β,β-dimethylacrylshikonin down-regulated the expression of XIAP, cIAP-2 and Bcl-2 and up-regulated the expression of Bak and Bax, and caused the loss of mitochondrial membrane potential and release of cytochrome c in SGC-7901 cells, consistent with mitochondria dependent apoptosis. The observation of β,β-dimethylacrylshikonin mediated activation of caspase-9, caspase-3, and subsequent cleavage of PARP, as well as the result that the pan-caspase inhibitor Z-VAD-FMK reduced β,β-dimethylacrylshikonin-induced apoptosis in SGC-7901 cells, suggesting that mitochondrial-mediated caspase cascade pathway plays a key role in β,β-dimethylacrylshikonin-induced apoptosis. Taken together, our results indicated that β,β-dimethylacrylshikonin regulates expression levels of apoptosis-related proteins, causes cytochrome c release and trigs caspase-dependent cell apoptotic death.

Mitogen-activated protein kinases (MAPK) regulate diverse cellular programs including embryogenesis, proliferation, differentiation and apoptosis [33]. The MAPK family proteins are composed of three protein kinases: ERK1/2(extracellular signal-regulated kinases 1 and 2), JNK (c-Jun N-terminal kinase) and p38 [34], [35]. In general, transient ERK activation leads to cell proliferation, but sustained activation of ERK is assoctiated with differentiation [36]. Emerging evidence suggests that the activation of ERK contributes to apoptosis. Jeong et al. showed that kaempferol caused cancer cell to undergo apoptosis through an ERK-dependent pathway [21], [37], [38]. Recently, it has been reported that icaritin induces growth inhibition and apoptosis in human PSMCs via ERK signaling pathway [39]. Li et al. demonstrated that activation of RAF/MEK/ERK signaling pathway plays a critical role in calcium-induced apoptosis of lens epithelial cells [40]. Here we also found that the sustained activation of ERK is involved in β,β-dimethylacrylshikonin-induced growth inhibition and apoptosis in SGC-7901 cells. U0126, a specific inhibitor of MEK (the MAPK/ERK kinase), effectively blocked β,β-dimethylacrylshikonin-induced ERK activation and attenuated β,β-dimethylacrylshikonin-induced apoptosis, suggesting the pro-apoptotic effects of β,β-dimethylacrylshikonin in SGC-7901 cells are mediated by the sustained activation of the ERK1/2 signaling pathway.

In conclusion, we found that β,β-dimethylacrylshikonin inhibited cell growth and induced apoptosis in SGC-7901 cells. We also studied the underlying mechanisms involved in β,β-dimethylacrylshikonin-induced apoptosis. Our results indicated that β,β-dimethylacrylshikonin-induced apoptosis involves in the reduction of XIAP, cIAP-2, and Bcl-2 protein expression and induction of Bak and Bax protein expression, and caused the loss of mitochondrial membrane potential and release of cytochrome c in SGC-7901 cells. Our results also demonstrated that β,β-dimethylacrylshikonin induced apoptosis involves the activation of caspase-3, caspase-8 and caspase-9 and the cleavage of PARP. Besides, ERK signaling pathway also participated in β,β-dimethylacrylshikonin-induced apoptosis. Our results indicated that β,β-dimethylacrylshikonin could be developed as a potential anticancer agent against human gastric cancer.
